# Teleoperation of Collaborative Robot for Remote Dementia Care in Home Environments

**DOI:** 10.1109/JTEHM.2020.3002384

**Published:** 2020-06-15

**Authors:** Honghao Lv, Geng Yang, Huiying Zhou, Xiaoyan Huang, Huayong Yang, Zhibo Pang

**Affiliations:** 1 State Key Laboratory of Fluid Power and Mechatronic Systems, School of Mechanical EngineeringZhejiang University 12377 Hangzhou310027China; 2 College of Electrical EngineeringZhejiang University 12377 Hangzhou310027China; 3 ABB Corporate Research 474050 72178VästeråsSweden

**Keywords:** Assistive robot, motion capture, remote dementia care, teleoperation, telerobotic system

## Abstract

As a senile chronic, progressive and currently incurable disease, dementia has an enormous impact on society and life quality of the elderly. The development of teleoperation technology has changed the traditional way of care delivery and brought a variety of novel applications for dementia care. In this paper, a telerobotic system is presented which gives the caregivers the capability of assisting dementia elderly remotely. The proposed system is composed of a dual-arm collaborative robot (YuMi) and a wearable motion capture device. The communication architecture is achieved by the robot operation system (ROS). The position-orientation data of the operator’s hand are obtained and used to control the YuMi robot. Besides, a path-constrained mapping method is designed for motion trajectory tracking between the robot and the operator in the progress of teleoperation. Meanwhile, corresponding experiments are conducted to verify the performance of the trajectory tracking using the path-constrained mapping method. Results show that the position tracking deviation between the trajectory of the operator and the robot measured by dynamic time warping distance is 1.05 mm at the sampling frequency of 7.5 Hz. Moreover, the practicability of the proposed system was verified by teleoperating the YuMi robot to pick up a medicine bottle and further demonstrated by assisting an elderly woman in picking up a cup remotely. The proposed telerobotic system has potential utility for improving the life quality of dementia elderly and the care effect of their caregivers.

## Graphical abstract

This research proposed a telerobotic system for remote dementia care based on inertial motion capture technology, which gives the caregivers the capability of assisting dementia elderly remotely.

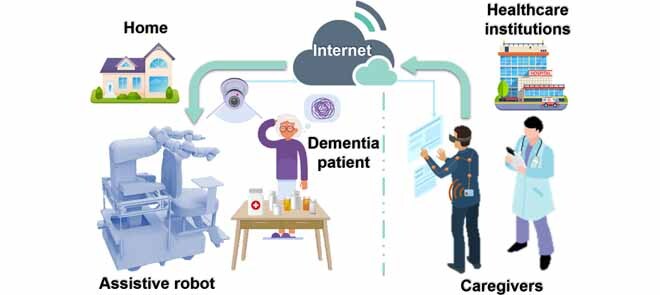

## I. Introduction

With the rapid population aging, more and more elderly people are suffering from dementia. Statistics indicate that one person develops dementia every 3 seconds in the world [1]. In parallel, global estimates of dementia prevalence are up to 7% of individuals above the age of 65 years [2]. Unfortunately, dementia disorders are chronic, long-lasting, progressive and currently incurable, which require coordinated care to address the medical, behavioral, and social aspects of the disease [3]. As a chronic disease, dementia has an enormous impact on both society and the life quality of patients. Long-term care at nursing homes or other healthcare institutions is a major component of this societal and economic burden [4]. In addition to such institutional care, informal caregivers (usually family members) provide a large proportion of dementia care [5].

For dementia elderly with obviously reduced capacity, supportive living situations and integrated care systems can give them a healthy life with dignity again [6]. However, healthcare services remain unmet, partly because of the insufficient number of caregivers. In order to make up for the shortage of healthcare staff, a promising trend in healthcare is to move the healthcare services from healthcare institutions to home [7]. It is also worth noting that these informal caregivers still experience psychological burden themselves due to the imbalance between their work and the care delivery [8], [9]. It is generally acknowledged that assistive technologies in home-based settings can promote the wellbeing of people with dementia, remedying any loss of autonomy for patients [10], and also give their caregiver periods of relief to lessen the burden on their shoulders [11].

In recent years, indoor assistive robots and integrated robotic systems for the elderly assistance have gradually come into people’s sight, and have been extensively used in the context of dementia care and management [12], [13]. Due to the limited mobility and degraded learning ability of the elderly with dementia, complex control methods make it impossible for them to control the robot on their own. This puts forward higher requirements for the intelligence and automation of assistive robots for dementia care [14]. To address this problem, engineers have moved toward the creation of generic robotic platforms that can be programmed to undertake various tasks that focus on improving the quality of life for older people with dementia [15]. Telerobotic systems that allow the caregivers to control the assistive robot remotely and conduct the telepresence of the elderly with dementia emerged under new circumstances. More detailed related works on assistive robots for remote dementia care are listed in Section II. However, Current research about the telerobotic technology for dementia care focus mainly on the monitoring of the elderly and most of the executive functions are simple and unreliable which limits their wide applications. Dementia care is a skilled and “operator-dependent” technique in which the caregivers need enough training and experiences [16]. In order to carry out the remote assistance, the novel solution of human-robot interaction that can accurately reflect the intention of the caregivers will have a great potential for remote dementia care [17]. Therefore, finding an effective and natural method to recognize the intention of the caregivers’ operation without imposing an additional burden for interaction with the assistive robots is one of the focus issues to be addressed for remote dementia care [18], [19].

**FIGURE 1.**
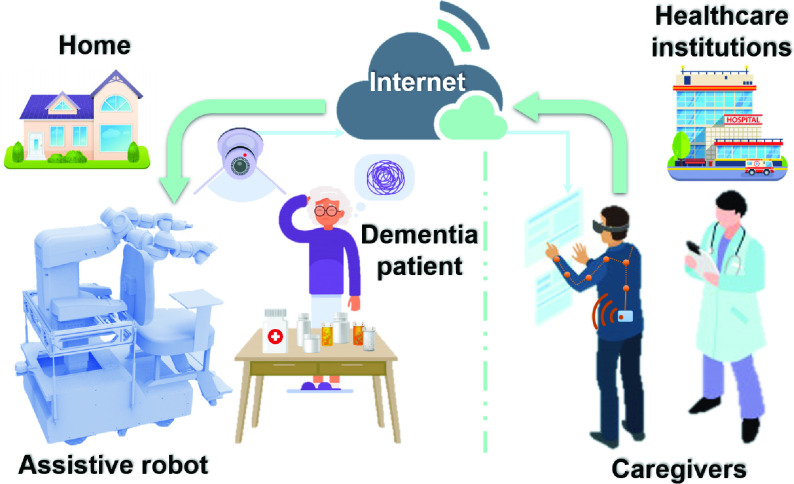
The overall architecture of the proposed telerobotic system for remote dementia care. (Vectors of the dementia patient and caregivers were designed by Freepik).

In this paper, we proposed a telerobotic system for remote dementia care based on inertial motion capture technology which is low training cost for caregivers and scalable for function realization. The diagram illustrating the framework of the whole system is shown in Fig. 1. A self-designed care-assistive robot is used in the proposed telerobotic system which can assist with mobility, household tasks [20]. The dual-arm collaborative robot YuMi with seven degrees of freedom (DOFs) of each arm is chosen as the manipulator during the teleoperation. During the teleoperation, the position-orientation data of the operator’s hand are obtained via coordinate transformations using the inertial motion capture system. Then the data will be transmitted to the industrial personal computer (IPC) installed with robot operating system (ROS) through the Internet [21]. Finally, the robot performs the corresponding action according to the operator’s intention to assist dementia elderly.

This work has the following primary contributions and novelties: 1) The design of a telerobotic system based on ROS for remote dementia care using inertial motion capture system; 2) The implementation of a path-constrained mapping method for teleoperation based on upper limb motion data of operator.

The remaining structure is as follows. Section II introduces the related work on assistive robots for remote dementia care, and the motivation of this work is given by concluding the pros and cons of current related studies. The architecture of the proposed system is introduced in Section III. In addition, Section III also details the approach of the motion capture and the motion mapping method between the operator and YuMi robot. Section IV describes the experimental settings and evaluates the performance of the proposed system with the analysis of the results of experiments. The verification and demonstration of picking up a medicine bottle and assisting an elderly woman using the telerobotic system are described in the last part of Section IV. Discussions and conclusions of this work are summarized in Section V.

## II. Related Work on Assistive Robots for Remote Dementia Care

Currently, many projects are exploiting assistive robots to support the aging life of elderly people. Assistive robots are autonomous robots that communicate with the users for a well-defined care purpose. Some assistive robots are used as assistive devices to support independent living and mobility. Others are used as companion robots, aiming at providing social support, increasing health and physiological well-being [22]. Significant research and development efforts have recently taken place for the design and development of assistive technologies to support the elderly in general and manage the dementia patients in specific.

### A. Functional Design of Current Assistive Robot

Pineau *et al.* from Carnegie Mellon University developed a mobile assistive robot named Nursebot, which can be used to assist elderly individuals with mild dementia and physical impairments, as well as support nurses in their daily activities. It also can provide companionship and help the elderly to communicate with physicians and caregivers remotely [23]. During experiments conducted in an assisted living facility, the Nursebot successfully demonstrated its ability to autonomously provide reminders and guidance for elderly residents. Besides, the ROBADOM project is devoted to the design of a robot-based solution for assisting activities of daily living: management of shopping lists, meetings, medicines for older adults [24]. The specificity of this project is to develop a specific robot for providing verbal and non-verbal help, support, and coaching during various tasks such as cognitive stimulation exercises. Meanwhile, the acceptance and the impact of the robot on older adults are addressed in the last step of the ROBADOM project. Also, an European Union (EU) funded project “HOBBIT– The Mutual Care Robot” was carried out and the Hobbit robot was designed to support household tasks, medical and social assistance for the elderly [25]. The Hobbit robot is also capable of fall prevention and detection as well as emergency detection and handling. Other included functions are: picking up objects from the floor, bring objects, offering reminders, and entertainment [26].

### B. Telerobotic Technology Applied on Assistive Care

The telerobotic technologies are usually employed in the telepresence robots for elderly assistive care, supporting better communication between patients with dementia and their caregivers. These robots are usually free-standing, wheel-based and feature a videoconferencing system that includes a web camera, moveable LCD screen, speaker, and microphone [15]. An important characteristic of such systems is mobility, which involves moving or steering the robot around the space, either from a remote location by a person or using space mapping functions embedded in an autonomously or semi-autonomously operated robot. For instance, one of the pioneer robots in this area was the Nursebot mentioned above in [23]. It uses natural language to provide information related to activities of daily living obtained from the web, and it also enables remote caregivers to establish telepresence at home. Another EU funded project GiraffPlus develops a telepresence robot, which allows relatives or caregivers to virtually visit an elderly at home [27]. GiraffPlus robot benefits from a network of sensors to monitor the activities in the home environment, placed in and around the home, as well as on the body of the elderly to extract high-level activities from sensor data [28], [29]. Furthermore, Puente and colleagues report a system enabling a mobile robot to autonomously pick-up objects a human is pointing at from the floor [30], and their proposed system is tested in real apartments with elderly people using the second prototype of the Hobbit robot mentioned in [25].

From the above description of the current related works, we can see that, on one hand, most researches of the assistive robots for dementia focus on companion and cognitive interaction ability. Telerobotic technology is mainly used in condition monitoring of the elderly [31]. On the other hand, only a few studies included caregivers other than the elderly or the patients. Different caregivers (e.g., nurses, family members, managers of nursing homes) have different needs and their expectations of robots vary widely, and it is important to consider their perspectives, so one of the starting points should be identification and examination of various caregivers’ expectations. The basic motivation of our work was given by the above pros and cons of the related studies, which consider the requests of patients, caregivers, and the application scenarios for dementia remote care.

## III. System Design and Implementation

### A. Overall Architecture

The proposed telerobotic system includes three main parts: caregivers as the tele-operator, assistive robot, and dementia elderly as the on-site patient. The tele-operator is the input of the assistive robot, i.e. caregivers such as family members and nursing staff not at home. The assistive robot is the receiver of the instruction and the output of the action. The on-site patient is a dementia elderly person who needs assistance and care.

**FIGURE 2.**
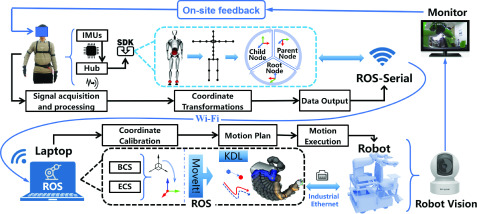
Detailed control block diagram of the proposed telerobotic system.

According to the detailed control block diagram of the system as shown in Fig. 2, the proposed telerobotic system can be divided into a human-motion-capture subsystem and a robot-control subsystem. The human-motion-capture subsystem obtains motion signals of the tele-operator through a wearable motion capture device. The dual-arm robot YuMi is chosen as the manipulator for carrying out the assistance of the robot-control subsystem [32]. This system is a multi-node distributed control system based on ROS, whose details are given below in section III.B and section III.C.

### B. Human-Motion-Capture Subsystem

#### 1) Basic Configuration

The motion capture system mainly consists of a wearable motion capture suit and the corresponding software, which can acquire, process, and transmit motion signals wirelessly in real time.

The motion capture suit, Perception Neuron, is a versatile and adaptable motion capture device which can be used both indoors and outdoors [33]. It consists of multi-node inertial measurement units (IMUs) which are composed of gyroscopes, accelerometers, and magnetometers. All IMUs are on straps which are worn on the body. Hub, the central processing unit of Perception Neuron, compiles and synchronizes motion data between IMUs and software through Wi-Fi. Axis Neuron, a supporting application of Perception Neuron in the Windows operating system (OS), is used to receive the motion data from the hub, then process and export the motion data of the wearer. Furthermore, we developed an executable program, called Neuron-to-ROS which can extract movement data from Axis Neuron and communicate with ROS through the serial port.

**FIGURE 3.**
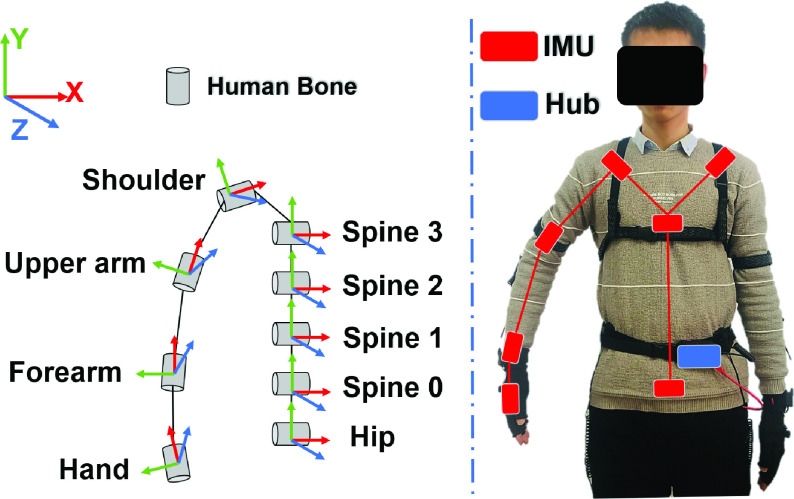
The skeleton model of the upper limb and the placement of IMUs on the human body. The skeleton model of the upper limb is structured using the processed data from the IMUs, in which the number of bones does not correspond to the number of IMUs.

As shown in Fig 3, the IMUs are mounted on straps which are worn on the upper limb. On the basis of the operator’s upper limb motion data acquired from the IMUs, the human skeleton model is constructed which is composed of the hip, spine 0, spine 1, spine 2, spine 3, shoulder, upper arm, forearm, right hand. In the process of motion mapping, the hip of the operator is set to coincide with the root node of the body’s skeleton model, while the right hand coincides with the end node. The other bones are matched with other nodes in turn according to the relative motion. According to the human skeleton model, the two adjacent nodes form a parent-child relationship, in which the node far away from the hip is the child node.

In addition, motion capture glove also belongs to the motion capture system, but the design and control of it are separated from the motion capture suit. The bending signals of the fingers, which can reflect the operator’s grab or release intentions, are transmitted to node of ROS through Wi-Fi to control the opening or closing state of the robot’s gripper. Since it is not the technical focus of this paper, the related details are not described here.

#### 2) Human Motion Tracking

**FIGURE 4.**
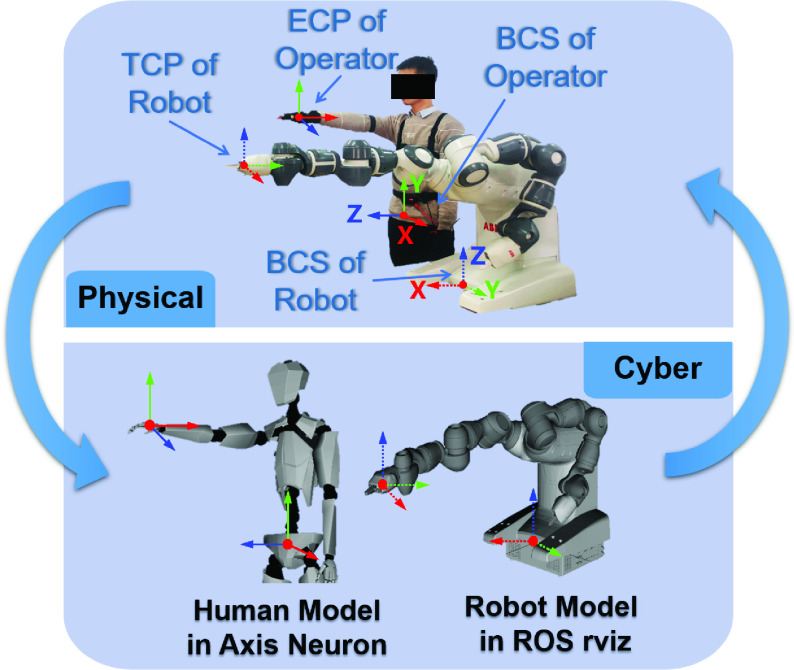
The cyber-physical architecture of the telerobotic system. The corresponding coordinate system of robot and operator is shown in the above, and the cyber model of the physical operator and robot is shown below.

During the human motion tracking, the motion capture device and ROS are synchronously connected to Neuron-to-ROS via TCP/IP protocol. The real-time maximum sampling frequency of the motion capture system is 120Hz. The Perception Neuron obtains the position-orientation data of wearer through IMUs which are mounted on the suit. Axis Neuron reflects human motion through a virtual animation model. However, because of different body sizes of wearers, the initial pose of the virtual animation model is not matched with the pose of human. Therefore, it is required to measure parameters of the body size such as arm length, shoulder width and the height of the wearer first. These parameters are set to create a corresponding virtual animation model. Then the motion calibration is performed to make the Axis Neuron display human motion synchronously through a virtual human model. The virtual animation model is shown in Fig. 4.

Besides, Axis Neuron exports motion data acquired from the motion capture suit to the Neuron-to-ROS in Windows Operation System (OS). Neuron-to-ROS, which can make connections between the Axis Neuron in Windows OS and ROS in Linux OS, is programmed to parse and transmit data streams based on the application program interface of Axis Neuron. The data streams include parameters of the human skeleton and motion data. The parameters describing the wearer’s skeleton size information are used to construct the corresponding human skeleton model. The motion data recording the limb motion include the position-orientation data of the human bones in the local coordinate systems. Neuron-to-ROS uses data streams to construct the human skeleton model and achieve coordinate transformations. Then the analytical position-orientation data of the human hand in the global coordinate system are published on ROS to control the robot.

### C. Robot-Control Subsystem

#### 1) Basic Configuration

Compared to the other robots, collaborative robot YuMi can ensure stop-motion in the presence of an operator or obstruction, and also can keep the electric motor drives on for a quick restart, which is crucial in the remote care operations. In addition, another research focus of our team is the development of robot skin for safe human-robot interaction which can provide host robots with the ability to detect much lower force to enhance safety performance [34]. Improving the safety in human-robot interaction by attaching flexible sensors to the robot’s arm is an ongoing work. The operation system of the YuMi robot, produced by Asea Brown Boveri Ltd. (ABB), is consistent with that of ABB industrial robot. The RobotWare OS running in the IRC5 controller is used to control the two arms of the YuMi robot. The single manipulator consists of a mechanical arm and a gripper, which is independently communicated with the main control board via industrial Ethernet.

In order to realize the distributed control based on ROS, we conducted the control framework utilizing ROS-Industrial. On the basis of the simple-message protocol, the motion control and socket communication of YuMi’s arms and grippers are configured in the application layer of the robot controller which is programmed in RAPID language. An IPC installed with ROS is used as the upper computer of the system. In the ROS interface layer, abb-driver, an official program package for the communication between ABB industrial robot and ROS, is configured. The motion planning and control of the robot are completed in MoveIt, an open-source project in ROS. In this case, we modified an open-source unified robot description format (URDF) model of YuMi provided by Lundell *et al.* [35]. Because YuMi’s manipulators and grippers are controlled independently based on different IP addresses, we divided the whole robot into four motion planning groups after URDF remodeling: left arm, right arm, left hand and right hand. In addition, the base coordinate system (BCS) of the YuMi robot is set in the front middle of the base in the URDF model.

#### 2) Motion Planning and Execution

In order to execute the movements of motion planning groups, the communication between IPC and YuMi is carried out via industrial Ethernet. The kinematics and dynamics library (KDL), a popular kinematics solver, is used to calculate the inverse kinematics and plan the movement of the robot’s motion planning group according to the start-state and goal-state of robot. The current position of the robot is chosen as the start-state of motion planning, and goal-state is determined by the specific position-orientation data of the operator’s hand in the process of the operation. The KDL solver calculates the robot’s joint values in the process and the motion is executed through MoveIt. Furthermore, in order to feedback the on-site status of the robot’s motion execution, a household web camera TL-IPC40C-4 is chosen to transmit the on-site video to the tele-operator. Bandwidth of the web camera is up to 300Mbps in the 2.4 GHz band.

The coordinate system of the operator is different from the coordinate system of the robot. According to the human skeleton model described above, the BCS of the tele-operator’s body is in the part of the hip. The coordinate system of hand is chosen as the end coordinate system (ECS), as shown in Fig. 4. Besides, the BCS of the robot is set in the front middle of its base, and the tool coordinate system is set in the middle of the robot’s gripper finger. The operator’s ECS position-orientation data obtained by the motion capture system cannot be used as the position-orientation data of the robot’s tool central point (TCP) directly to conduct the robot control. It is important to note that the coordinate system based on the wearable device is different from the traditional coordinate system. Therefore, we need to adjust YuMi robot’s model for making coordinate system corresponding in the ROS node program based on the above coordinate transformations. The orientation offset $\delta O$ between ECS and TCP is set to zero by modifying the URDF model of YuMi robot.

Based on the point-point mapping method, we proposed a novel path-constrained mapping method for teleoperation, which is more applicable for the continuous trajectory following. The path-constrained mapping method is based on trajectory pre-sampling in periodic time intervals to achieve the robot control. With a pre-defined time interval $\Delta T$, a series of operator’s ECS position-orientation data [N_P($t-\Delta T-\delta t$: $t$), N_O($t-\Delta T-\delta t$: $t$)] and current TCP pose data [R_P($t$), R_O($t$)] are input as the path-constrained points for robot’s motion planning. $\delta t$ is the refresh time of robot motion planning in ROS. A series of points [R_P($t+\delta t$: $t+\Delta T+\delta t$), R_O($t+\delta t$: $t+\Delta T+\delta t$)] are output as the robot’s TCP pose data within the next sampling time $\Delta T$. The detailed procedure is described in Algorithm 1.

Algorithm 1 **Path-Constrained Mapping Method**
Input: [N_P($t-\Delta T-\delta t$: $t$), N_O($t-\Delta T-\delta t$: $t$)], [R_P($t$), R_O($t$)]
Require: $\delta O =0$
Output: [R_P($t+\delta t$: $t+\Delta T+\delta t$), R_O($t+\delta t$: $t+\Delta T+\delta t$)]
1: R_P($t-\Delta T-\delta t$: $t) =$ N_P($t-\Delta T-\delta t$: $t) + \delta P$
2: R_O($\text{t}-\Delta T-\delta t$: $t) =$ N_O ($t-\Delta T-\delta t$: $t$)
3: Waypoints = ([R_P ($t$), R_O($t$)])
4: For $i = t-\Delta T-\delta t$: $t$
5: Waypoints = Add(Waypoints, [N_P($i$), N_O($i$)])
6: End for
7: Traj = MoveitPlan(Waypoints)
8: For $i = t+\delta t$: $t+\Delta T+\delta t$
9: [R_P($i$), R_O($i$)] = Sampling(Traj)
10: End for
11: Clear Waypoints

## IV. Experiments and Results

### A. Experimental Setting

In this experiment, the operator carried out the teleoperation using the proposed path-constrained mapping method. The rosbag, a gadget in ROS, was used for recording the real-time position-orientation data of the operator’s ECS and robot’s TCP during the experiments. Before carrying out this research, approval of the Ethics Committee of the 117th Hospital of People’s Liberation Army (PLA) has been obtained.

At first, a wearable motion capture suit was worn by the operator with a USB data wire connected to the laptop. The IP address and port number of Axis Neuron for data transmission needed to be set, then the data wire between the wearable device and laptop was disconnected and the two devices reconnected through Wi-Fi. After reconnection, the motion calibration process was performed, and the motion data of operator were transmitted to the software terminal in real time. On the robot side, the YuMi robot was turned on first and the background program configured on robot controller started through the FlexPendant for communication with ROS. The coordinate calibration mentioned above was conducted for motion mapping between the tele-operator and robot. Then ROS nodes for communication between the motion capture system and the robot started up for receiving real-time motion data of operator to control the robot. The movement speed of the robot’s end effector was set to 0.135m/s. $\Delta T$ and $\delta t$ were configured as 1 second and 0.2 seconds respectively.

Once the experiment settings above were completed, the operator moved his upper limb and the robot’s arm moved following the operator’s motion. The details of the experiments and the analysis of the results are as follows.

### B. Experiments Results and Analysis

In order to verify the tracking performance of the robot’s motion trajectory when using the path-constrained mapping method, we designed trajectory tracking experiments of four pre-defined trajectories which cover the basic spatial characteristics in cartesian coordinate space: horizontal line “—”, vertical line “$\vert $”, triangle “$\Delta $”, and arc “$\frown $”. All trajectories were performed by the operator at five pre-sampling frequencies to study the influence of sampling frequency on the performance of trajectory tracking.

**FIGURE 5.**
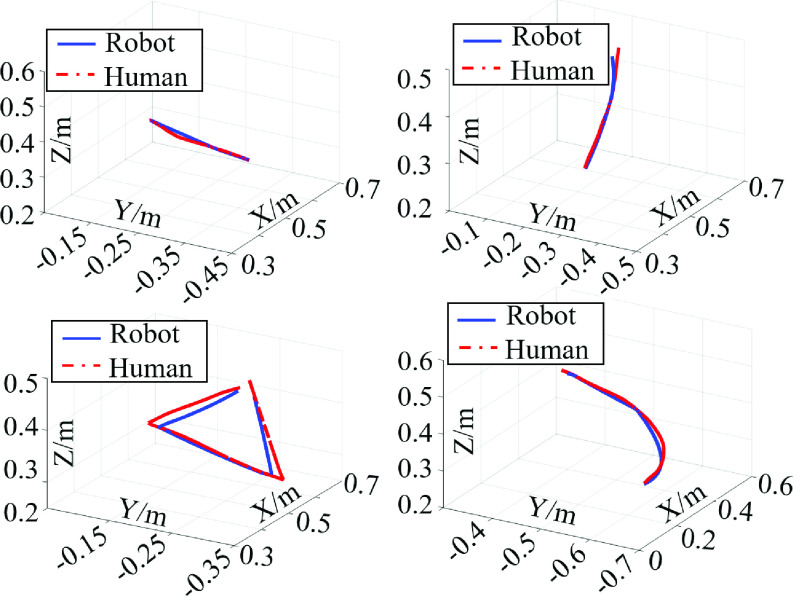
The tracking performance of the four trajectories (horizontal line “—”, vertical line “$\vert $”, triangle “$\Delta $”, and arc “$\frown $”) at the sampling frequency of 7.5Hz. The blue lines refer to the tracking trajectories of the robot, while the red dotted lines refer to the hand trajectories of the operator.

The operator performed the four trajectories “—” “$\vert $” “$\Delta $” “$\frown $” and the robot moved following the hand trajectory of the operator. The tracking experiment of each trajectory was repeated five times to ensure the reliability of the data. As shown in Fig. 5, the four trajectories at the pre-sampling frequency of 7.5Hz were displayed. Because the robot’s motion speed was different from the operator’s motion speed, the delay was inevitable between the robot trajectory and operator’s hand trajectory. The sampling points of planned trajectory and the tracked trajectory were not one-to-one correspondence at the same moment. In order to measure the tracking performance of motion trajectories between robot and operator, the dynamic time warping (DTW) algorithm was used to decrease the distortion of trajectory signals in the time domain [36], [37]. The DTW distance warped the time series first and then calculated the distance between two trajectories. Essentially, the calculated DTW distance was the absolute Euclidean distance.

After obtaining the trajectory data, the DTW distances between the hand trajectory of operator and the tracking trajectory of robot were calculated using the DTW algorithm. The DTW distances of the position data ($x$, $y$, $z$) and the orientation data (*roll*, *pitch*, *yaw*) between the two trajectories are shown in Table 1. The four trajectories had different spatial characteristics: (1) the coordinate change of trajectory horizontal line “—” occurred mainly along the Y-axis; (2) the coordinate change of vertical line “$\vert $” occurred mainly along the Z-axis; (3) the coordinate change of triangle “$\Delta $” occurred mainly along the Y-axis and Z-axis; (4) the coordinate change of arc “$\frown $” occurred mainly along the X-axis and Y-axis. Theoretically, the maximum error appeared in the direction where the coordinate changed most in the trajectory. At a high sampling frequency, the experimental results fully conformed to the theoretical inference. The abnormal result at the lower 2 Hz sampling frequency was due to the too few sampling points which result in a large mapping deviation and abnormal error distribution.

**TABLE 1:** The average DTW distances of the four trajectories.

Freq		2.0Hz					3.5Hz					5.0Hz					7.5Hz					10.0Hz				
Type		—	$\vert $	$\Delta$	$\frown $	Mean	—	$\vert $	$\Delta$	$\frown $	Mean	—	$\vert $	$\Delta$	$\frown $	Mean	—	$\vert $	$\Delta$	$\frown $	Mean	—	$\vert $	$\Delta$	$\frown $	Mean
Position	$\boldsymbol {x}$^a^	28.71	2.80	2.79	66.90	25.30	0.50	0.12	13.62	0.70	3.74	0.01	0.14	1.06	11.39	3.15	0.01	1.09	0.31	0.64	0.51	0.01	0.01	0.40	2.40	0.71
	$\boldsymbol {y}$	0.04	0.82	18.63	12.22	7.93	0.32	0.00	65.71	1.32	16.84	0.16	0.17	30.00	0.61	7.73	0.21	0.12	4.60	0.52	1.36	0.49	0.01	10.26	1.07	2.96
	$\boldsymbol {z}$	17.35	7.25	44.10	1.03	17.43	0.35	0.10	5.74	0.03	1.55	0.01	0.31	3.18	4.35	1.96	0.02	0.45	1.73	2.11	1.08	0.05	0.11	1.15	1.32	0.66
	RMS	19.37	4.51	27.69	39.27	18.32	0.40	0.09	38.88	0.86	10.00	0.09	0.22	17.43	7.05	4.95	0.12	0.69	2.84	1.31	1.05	0.29	0.06	5.97	1.70	1.80
Orientation	roll^b^	0.11	0.04	0.11	0.00	0.06	0.00	0.00	0.04	0.00	0.01	0.00	0.00	0.01	0.01	0.01	0.00	0.00	0.00	0.01	0.00	0.00	0.00	0.00	0.00	0.00
	pitch	1.27	0.00	0.04	0.00	0.33	0.02	0.01	0.24	0.00	0.07	0.00	0.02	0.00	0.17	0.05	0.00	0.01	0.01	0.00	0.01	0.00	0.00	0.00	0.00	0.00
	yaw	0.36	0.00	0.06	0.24	0.17	0.00	0.00	0.39	0.00	0.10	0.00	0.02	0.11	0.02	0.04	0.00	0.01	0.03	0.00	0.01	0.00	0.00	0.03	0.01	0.01
	RMS	0.76	0.00	0.04	0.15	0.21	0.01	0.00	0.26	0.00	0.07	0.00	0.01	0.06	0.10	0.03	0.00	0.01	0.03	0.01	0.01	0.00	0.00	0.03	0.02	0.01

^a^ The unit of the position parameters ($x$, $y$, $z$)’s DTW distance in TABLE I is set as millimeter (mm.), to facilitate the presentation of the data.

^b^ The unit of the orientation parameters (roll, pitch, yaw)’s DTW distance in TABLE I is set as radian (rad).

**FIGURE 6.**
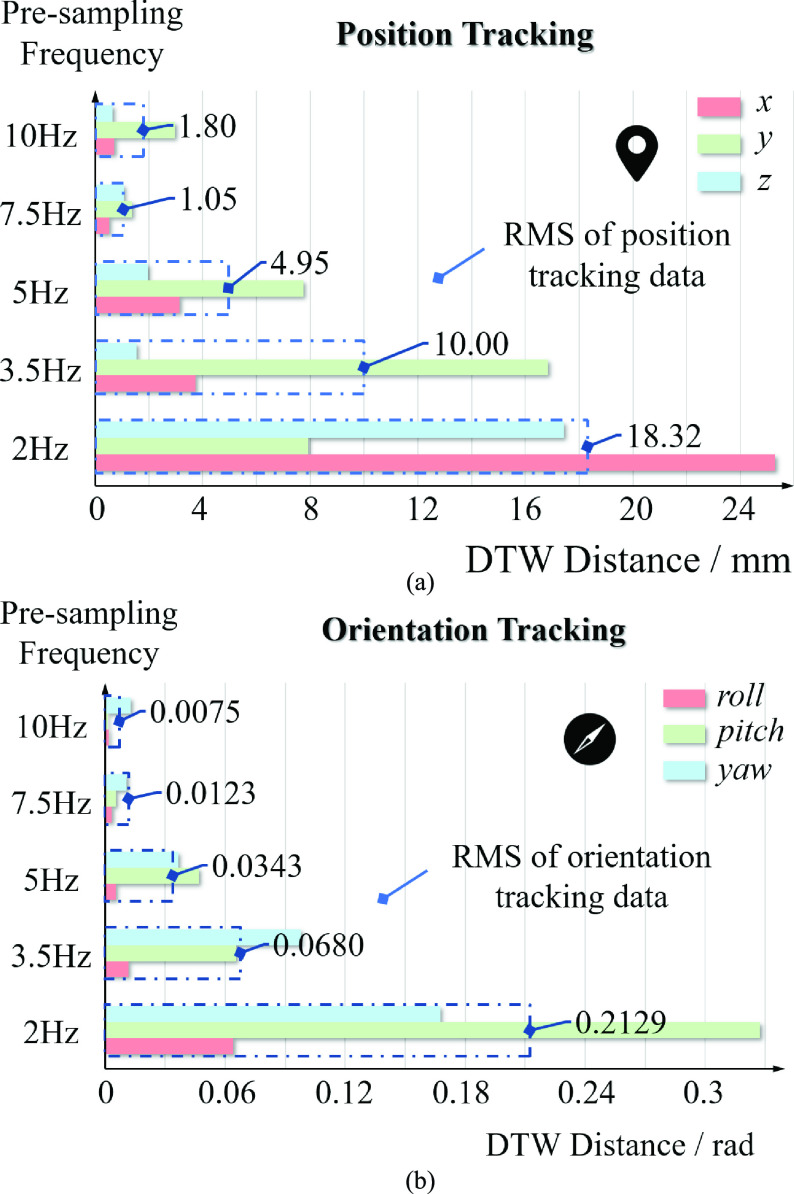
Overview of the tracking performance by DTW distance measure. (a) position tracking performance at five sampling frequencies; (b) orientation tracking performance at five sampling frequencies.

For a more comprehensive evaluation of the tracking performance, we calculated the root mean square (RMS) of position data and orientation data respectively. As shown in Table 1, the DTW distances of the corresponding trajectories degraded with the increase of pre-sampling frequency, which meant the increasing tracking performance between the trajectories of human and robot, both position and orientation. The DTW distances at 7.5 Hz and 10.0 Hz were no more than 2.00 mm. However, the DTW distance at 10.0 Hz was 1.80 mm, which was larger than the 1.05 mm at 7.5 Hz. This was due to the excessive number of sampling points at 10.0 Hz, which led to more complex motion planning and the decreased tracking performance of the robot’s motion trajectory. About the orientation tracking performance, the smallest orientation DTW distance was achieved at the frequency of 10.0 Hz, not 7.5 Hz, with the average orientation DTW distance of 0.0075 rad. The reason was that the orientation of the operator’s hand changed a bit during the experiment. In order to show the data of tracking performance more intuitively, we gave a graphical representation of the experiments results, as shown in Fig. 6.

### C. Verification and Demonstration

**FIGURE 7.**
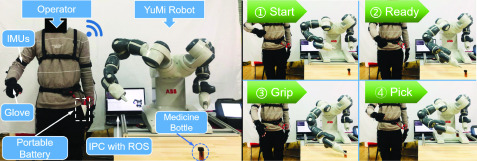
Demonstration of picking up a medicine bottle using the proposed telerobotic system.

The proposed telerobotic system had great potential in the home environment for remote dementia care. It made it possible that the caregivers assist the elderly remotely to do some essential chores that are difficult by themselves, such as assisting them to get the medicine correctly. In order to conduct the verification of the practical application performance of the proposed telerobotic system for remote dementia care, we used the proposed telerobotic system to conduct a task of picking up a medicine bottle at first, and the operational process is displayed in Fig. 7.

In this application, the operator put on the wearable motion capture device first. Then the operator performed the corresponding picking action by the side of the robot. The motion capture device was powered by a portable battery, and the motion data were processed and transmitted to IPC installed with ROS through Wi-Fi. Then the robot was controlled using the obtained motion data. The process was manually divided into four processes: Start, Ready, Grip Pick, as shown in Fig. 7. The operator started from the starting position and moved his hand to a posture ready to pick. Then the robot moved its gripper following the operator’s upper limb motion. When the gripper moved to a position that is suitable for picking, the operator spread his hand to open the robot’s gripper and picked up the medicine bottle.

**FIGURE 8.**
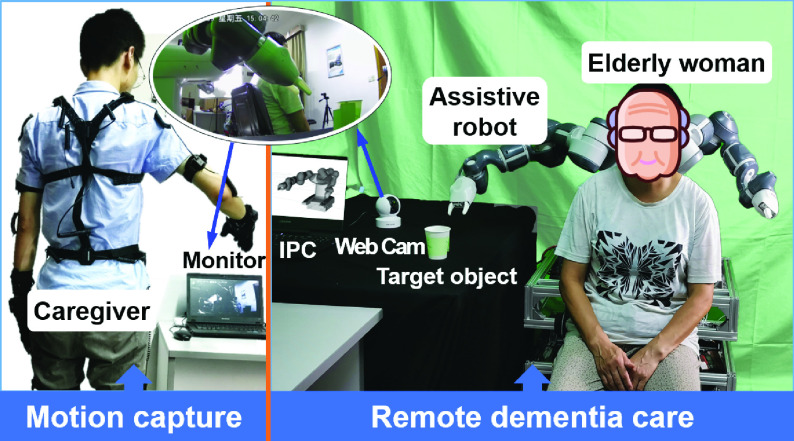
Demonstration of assisting an elderly woman in picking up a cup using the proposed telerobotic system remotely.

The functional performance of the proposed system has been reflected through the above application. In order to demonstrate the practicability of the proposed system for remote dementia care, we further applied the proposed telerobotic system to conduct a task of assisting a remote elderly woman in picking up necessaries, and a cup was demonstrated as an example here. The implementation of this application is demonstrated in Fig. 8. Further, acceptability of the assistive robot and the reaction of the care recipient merited consideration. The appearance design of YuMi’s dual-arm was anthropomorphic, and the ultra-light magnesium arm rotated on seven axes to mimic human-like movements with greater agility than 6-axis robots. The above features of YuMi robot were friendly and acceptable to the care recipient during the teleoperation progress.

In the second application scenario, the care recipient and the caregiver stayed into two different rooms respectively. The caregiver put on the wearable device for motion capture at first. On-site video of the remote elderly woman was captured through a web camera and transmitted to the monitor in front of the caregiver through the Internet for teleoperation. The delay existed using the web camera for feedback compared to the first application scenario. The caregiver performed the corresponding picking action and adjusted according to the video from the remote scene. The motion data were used for planning the robot’s motion path in the IPC which was connected to the robot based on industrial Ethernet through a wireless bridge. Then the robot was controlled using the obtained data remotely.

The elderly woman sat on the seat of our self-designed nursing-care robot waiting for assistance. More details of the self-designed care-assistive robot can be found in [20]. The robot moved its arm following the remote operator’s upper limb motion until its gripper had arrived at a suitable place for grip according to the on-site feedback. When the gripper moved to a position that is suitable for picking, the operator spread his hand to open the robot’s gripper and then the robot picked up the cup. Then the caregiver delivered the cup to where the elderly woman can get it easily on the premise of ensuring safety. At last, the elderly woman got the cup easily from the robot based on the telerobotic system.

## V. Discussion and Conclusion

In this paper, a novel telerobotic system composed of a wearable inertial motion capture device and a dual-arm collaborative robot for remote dementia care is proposed. Based on the upper limb motion data obtained from the motion capture system, the position-orientation data of the operator’s hand are acquired via coordinate transformations. The control of the robot is realized by mapping movements from the upper limb of the tele-operator to the YuMi robot according to the position-orientation data of the operator’s hand. In addition, a path-constrained mapping method is proposed for better performance of trajectory tracking.

The performance of the proposed telerobotic system using the path-constrained mapping method is evaluated by comparing the trajectory tracking deviations of four pre-defined trajectories under five sampling frequencies. As a result, based on the path-constrained mapping method, the position tracking deviation between the trajectory of operator and robot measured by DTW distance is 1.05 mm at the sampling frequency of 7.5 Hz, which is the best tracking performance in the experiments. Furthermore, the system is demonstrated by teleoperating the robot to pick up a medicine bottle and assisting an elderly woman to get a cup, which further demonstrates the practicability of the proposed system for carrying out the assistance for remote dementia care.

It is undeniable that there are still some limitations in the current work. Future work will focus on dealing with the time delay of the path-constrained mapping method. In addition, the dual-arm teleoperation and the collaborative control strategy during the teleoperation tasks will be also considered in the future works [38]. Under the configuration of the current web camera, the delay time is within the tolerance of normal operation and the caregivers can complete the teleoperation slowly. In the future works, a feedback solution with better transmission performance will be considered to get the lower latency.
